# Supplementary material for: The Characteristics, Distribution, Function, and Origin of Alternative Lateral Horse Gaits
Source: Animals (Basel). 2023 Aug 8;13(16):2557. doi: 10.3390/ani13162557 (PMC10451235; doi:10.3390/ani13162557)
Supplement: Supplementary file 1 [file animals-13-02557-s001.zip › animals-2481173-supplementary.pdf]

Supplementary Material

Table S1: Table showing key temporal kinematic, linear kinematic, biomechanical, and angular variables for the different horse gaits (i.e. walk, flat walk, running walk, fox walk, broken trot, trot, rack, broken pace, hard pace, canter, and gallop) [Sources of data given in references below].

Gait	Walk	Lateral Flat Walk (Flat-Foot(ed) Walk)	Running Walk	Diagonal Flat Walk (Fox Walk; Dog Walk)	Fox Trot (Broken Trot)	Trot (Flying Trot)	Rack (Tölt; Singlefoot)	Stepping Pace (Amble; Broken Pace)	Pace (Flying Pace; Hard Pace)
Beats (audible)	Four	Four	Four	Four	Four	Two	Four	Four	Two
Sequence (temporal)	Lateral	Lateral	Lateral	Lateral	Lateral	Diagonal	Lateral	Lateral	Lateral
Couplets (temporal)	None<br><br>Isolated(single-foot)	Lateral to Isolated (single-foot) [Ideal]	Lateral to Isolated (single-foot) [Ideal]	Diagonal to Isolated (single-foot) [Ideal]	Diagonal	Diagonal	Lateral to Isolated (single-foot) [Ideal]		
Chronicity<br><br>Audible Step Time Ratio	Even<br><br>(0.80-0.93)	Uneven to Even (Ideal)<br><br>(0.63-0.67)	Uneven to Even (Ideal)<br>(0.62-0.88)	Uneven to Even [Ideal]<br><br>(0.94)	Uneven [Shuffled]<br><br>(0.17-0.48)	Even<br><br>(0.84-0.97)	Uneven to Even<br><br>(0.70-0.92)	Uneven [Shuffled]<br><br>(0.36-0.54;	Even<br><br>(0.74-0.89)
Biomechanics	Inverted Pendulum	Inverted Pendulum; Hind Leg Outstretch and Push	Inverted Pendulum; Hind Leg Outstretch and Push	Inverted Pendulum; Hind Leg Outstretch and Push	Spring-Mass	Spring-Mass	Spring-Mass	Spring-Mass	Spring-Mass
Velocity (meters/ second)	1.40-1.80	1.80-2.20	2.20-4.00	1.80	3.20-3.70	2.50-6.50<br><br>(8.5-12.0 racing)	2.50-5.98<br><br>(10.6 racing)	2.90-6.20	3.50-8.10<br><br>(10.5-14.0 racing)
Stride Duration (seconds)	1.30-1.00	1.10-0.95	0.85-0.65	1.15-1.20	0.67-0.58	0.80-0.56	0.70-0.42	0.61-0.45	0.60-0.30

						(0.55-0.46 racing)			
Front Stance/ Stride Duration (%)	0.68-0.59	0.61-0.57	0.51-0.46	0.57	0.57-0.48	0.66-0.27	0.59-0.34	0.54-0.38	0.44-0.28
Hind Stance/ Stride Duration [Duty Factor] (%)	0.69-0.57	0.59-0.61	0.58-0.52	---	0.59-0.55	0.58-0.27	0.66-0.32	0.55-0.41	0.46-0.28
Overlap of Ipsilateral Limbs in Swing Phase (%)	0.33-0.39	0.32-0.35	0.52-0.70	---	0.20-0.40	0.04-0.13	0.50-0.70	0.68-0.89	0.76-0.99
Lateral Advanced Placement [Limb Phasing Value] (% of stride duration)	0.25-0.22	0.20-0.18	0.23-0.17	0.30	0.41-0.32	0.49-0.44	0.24-0.18	0.18-0.13	0.10-0.05
Lateral Advanced Liftoff (% of stride duration)	0.25- 0.24	0.23-0.21	0.19-0.10	----	0.36-0.33	0.49-0.46	0.22-0.10	0.16-0.10	0.12-0.07
Diagonal Advanced Placement (% of stride duration)	0.28-0.25	0.30-0.29	0.35-0.27	0.32	0.20-0.07	0.06-0.01	0.34-0.24	0.37-0.33	0.45-0.40
Diagonal Advanced Liftoff (% of stride duration)	0.27-0.25	0.31-0.27	0.48-0.31	---	0.19-0.14	0.04-0.01	0.35-0.26	0.44-0.34	0.50-0.34
Stride Length (meters)	1.40-1.90	1.80-2.40	2.10-2.90	1.95	2.00-2.50	2.00-3.80<br>(4.50-6.00 racing)	1.80-3.20<br>(4.15-4.60 racing)	2.00-3.10	2.10-3.50<br>(5.40-6.30 racing)
Overstep Length (cm)	-4 to 25	28-45	36-72	---	13-17	-21 to 39<br>(193 racing)	26-101	67-90	71-77<br>(153 racing)
Average Interior Straddle (Range) (cm)	2.6<br>(0,0-6.4)	-0.2<br>(-1.0 to 1.0)	0.1<br>(-0.7 to 2.5)	---	ca. 8.3<br>(3.8-11.4)	1.9<br>(0.0-6.4)	-2.9<br>(-8.9 to 5.8)	-2.5<br>(-3.9 to 2.0)	1.4<br>(-2.3 to 5.0

Overstep Length/Stride Length (ratio)	-0.03 to 0.14	0.14-0.23	0.18-0.28	---	0.05-0.09	-0.11 to 0.11 (0.30-0.35 racing)	0.16-0.45	0.31-0.41	0.26-0.37
Head Vertical Displacement (Nod) (cm)	2.0-10.5	11.0-18.5	10.0-24.0	15.0	12.0	6.0-8.0	5.5-7.5	ca. 6.0	5.0
Shoulders (Withers; Sternum) Vertical Displacement (cm)	2.7-3.6	8.2-10.7 cm	---	---	---	4.7-9.2	5.4-7.0	ca. 4.0-4.7	ca. 4.0-4.7
Center of Mass (Saddle; Loin) Vertical Displacement (cm)	ca. 3.0-5.9	7.5-9.3	---	---	---	5.3-10.3	1.2-5.2	---	---
Croup (Sacrum) Vertical Displacement (cm)	3.0-7.0	8.0-11.0	ca. 3.0-6.0	---	ca. 4.0-8.0	5.0-13.0	3.0-6.5	ca. 4.0	4.0-4.5
Front Leg Protraction/Retraction Angles (degrees from perpendicular line at scapula)	64.6-72.3/ 102.4-116.1	---/---	ca. 65.0-75.0/ ca. 110.0-120.0	68.0/ 125.0	62.0/ 126.0	63.7-72.7/ 101.0-115.0	68.2-71.2/ 112.0-120.2	ca. 70.0/ 115.0	ca. 50.0-57.0/ ca. 125.0-130.0
Hind Leg Protraction/Retraction Angles (degrees)	66.9-71.2/ 107.5-113.6	ca. 60.0-70.0/ ca. 110.0-115.0	ca. 55.0-65.0/ ca. 120.0	---/---	66.9-68.6/ 106.5-108.1	65.5-80.0/ 106.9-120.0	64.9-67.2/ 105.4-106.7	ca. 64.0-68.0/ ca. 110.0-113.0	ca. 50.0-55.0/ ca. 118.0-130.0
Shoulder Angle Midstance/ Elevated (degrees)	113.1-119.2/ 119.2-126.2	101.3-101.5/ 108.5-108.8	---/---	103.0/110.0	100.0-104.0/ 113.0-120.0	122.7-125.4/ 110.1-115.0	---/---	---/---	---/---
Hip Angle Midstance/Elevated (degrees)	111.1-111.3/ 86.3-107.7	100.9-111.5/ 80.1-88.2	---/---	---/---	---/---	100.0-112.2/ 71.3-88.9	---/---	---/---	---/---
Elbow Angle Midstance/Elevated (degrees)	145.3-156.6/ 104.8-109.0	151.3-154.3/ 97.0-108.5	ca. 140.0-150.0/	151.0/99.0	140.0-149.0/ 97.0-115.0	155.5-166.1/ 79.3-95.3	ca. 145.0/ 88.8-90.3	ca. 140.0-150.0/	ca. 150.0/ ca. 90.0

			ca. 105.0-115.0					ca. 90.0-115.0	
Stifle Angle Midstance/Elevated (degrees)	159.7-173.0/ 130.6-141.0	152.8-157.3/ 115.7-124.0	ca. 140.0-150.0/ca. 125.0-130.0	---/---	ca. 135.0-140.0-/ca. 115.0-120.0	150.0-169.0/ 105.0-121.7	ca. 147.0-155.0-/105.4-112.6	ca. 135.0-140.0/ ca. 105.0-125.0	ca. 135.0-/ ca. 115.0
Knee (Carpal) Midstance/Elevated (degrees)	170.0-180.5/ 104.8-112.2	184.0-184.9/ 128.4-131.4	ca. 178.0-189.0 /ca. 120.0-127.0	185.0/147.0	180.0-184.0/ 130.0-140.0	176.5-194.8/ 92.1-112.9	ca. 172.0-180.0/ 119.4-122.4	ca. 180.0-190.0/ ca. 90.0-120.0	ca. 175.0-185.0/ ca. 85.0-100.0
Hock (Tarsus) Midstance/Elevated (degrees)	154.0-169.6/ 127.5-134.0	163.1-167.0/ 121.8-127.0	ca. 130.0-144.0/ ca. 95.0-120.0	---/---	144.7-145.6/ 86.8-90.8	151.6-169.7/ 96.7-114.3	ca. 135.0-145.0/ 93.0	ca. 135.0-155.0/ ca. 107.0-110.0	ca. 145.0/ ca. 80.0-95.0
Front Fetlock Midstance/Elevated (degrees)	220.0-221.1/ 160.0-177.9	221.8-222.2/ 165.5-175.4	240.0-242.0/ 176.0-177.0	224.0/155.0	229.0-230.0/ 139.7-160.0	215.0-243.7/ 134.1 -158.7	235.9-236.6/ 133.4-144.5	ca. 215.0-240.0/ ca. 125.0-145.0	ca. 260.0/ ca. 135.0-160.0
Hind Fetlock Midstance/Elevated (degrees)	202.0-222.9/ 143.0-147.7	214.8-216.2/ 151.2-156.0	ca. 225.0-242.0/ ca. 140.0-155.0	---/---	240.9-241.4/ 151.6-154.9	210-233.0/ 114.3-128.6	236.1-236.3/ 151.1-153.0	ca. 240.0-245.0/ ca. 140.0-145.0	ca. 265.0/ ca. 140.0
Quadrupedal Support (%)	0	0	0	0	0-7	0	0	0	0
Tripedal Support (2F/2H) (%)	19-53 (9-25/ 10-28)	38-68 (18-33/ 20-35)	5-23 (0-7/ 5-16)	---	10-29 (5-15/ 5-14)	0	4-37 (2-16/ 2-21)	4 (2/2)	5-10 (2-4/ 3-6)
Lateral Bipedal Support	10-27	23-43	50-67	--	11-33	0-4	22-60	62-72	74-90
Diagonal Bipedal Support	9-20	9-19	16-35	---	52-71	57-75	8-39	6-32	0-21
Single Leg Support (Front/ Hind)	0	0	2-8 (0/2-8)	0	0	8-20 (0/8-20)	0-64 (0-26/ 0-38)	0-27 (0/0-27)	0-13 (0-7/ 0-6)
Four-legged Suspension (%)	0	0	0	0	0	6-22	0	0	7-25

Table S2: Demographics of North American Gaited Horses.

NORTH AMERICA											
Breed	Number of Individuals	Country	Region	Zone	Year Breed Originated<br><br>[Registry Founded]	Gait(s)	A-allele [AA+CA genotypes]	AA genotype	CA genotype	CC genotype	Reference
American Paso Fino [PU]<br><br>[Cross of Puerto Rican Paso Fino + Colombian Paso Fino]	ca. 500	United States	South Carolina, Texas	Riding Horse	[1964]	rack [saddle rack, named classic fino; paso corto; paso largo; broken trot [paso corto; paso largo]; broken pace [andadura, not ideal]	0.96<br><br>[1.00]	0.91	0.09	0.00	Promerová et al., 2014
American Saddlebred [SB] [American Saddle Horse; Kentucky Saddler; Golden American Saddlebred; National Saddle Horse]	75,000	United States	Kentucky; Montana (1979)	Bluegrass Hills; Riding Horse	ca. 1839<br><br>[1891; 1957]	[some]<br><br>rack [saddle rack (medium) or hard rack (fast)]; broken pace [slow gait (slow)]	0.28<br><br>[0.48]<br><br>0.25<br><br>[0.41]	0.07<br><br>0.10	0.41<br><br>0.31	0.52<br><br>0.59	Promerová et al., 2014; Regatieri et al., 2015;<br><br>Gaspar et al., 2021
American Walking Pony [WP] [American Gaited Pony]	ca. 100	United States	Georgia	Riding Horse	1968	running walk [pleasure walk (slow); merry	---	---	---	---	---

[Cross of Tennessee Walking Horse + Welsh Pony]						walk (fast)<br>[some] rack [saddle rack to hard rack]; broken pace [slow gait]					
Appalachian Singlefoot Horse [AS]	ca. 50	United States	North Carolina, Appalachians	Appalachian Mountains; Riding Horse	1981	broken trot [singlefoot]	---	---	---	---	---
Appaloosa [AP] [Cayuse; Elegesi Qiyus; Nez Perce; Palouse]	525,000	United States; Canada	Oregon and Washington States, Columbia Plateau; Porterville, California; Nemaiah Valley, British Columbia, Canada	Columbia Plateau, Riding Horse	ca. 1710<br>[1938; 1961]	[some]<br>broken pace [Indian shuffle; Appaloosa shuffle]	0.13<br>[0.20]<br>0.09<br>[0.16]	0.05<br>0.01	0.15<br>0.15	0.80<br>0.84	Pomerová et al., 2014<br>Gaspar et al., 2021
Canadian Pacer [CP] [Dutch Horse; Old Canadian Horse]	0	Canada	Quebec	Taiga and Boreal Shields; Riding Horse	1665; 1812-1886<br>[Extinct]	pace	---	---	---	---	---
Canadian Pony of the Americas [PA] [Kanata Pony]	50,000	United States; Canada	Indiana and Iowa, Mason City; British Columbia	Riding Horse	1954<br>[1975]	[some]<br>running walk [flat walk (slow); hard running	0.18<br>[0.30]	0.06	0.24	0.70	Gaspar et al., 2021

[Cross of Appaloosa + Shetland Pony]						walk (fast)]					
Carolina Marsh Tacky [MT]	300	United States	South Carolina, Lowcountry, Beaufort, Berkeley, Charleston, Colleton, Dorchester, Georgetown, Hampton, Jasper, and Williamsburg Counties	Coastal Marshes	1565 [2007]	broken trot [rocking chair trot; swamp trot]	---	---	---	---	---
Florida Cracker Horse [FC] [Chickasaw Pony; Cherokee Pony; Choctaw Pony; Florida Cow Pony; Florida Horse; Grass Gut; Indian Pony; Prairie Pony; Seminole Pony]	1300	United States	Florida, Alachua, Paynes Prairie ; Mississippi ; Kiamichi Mountains, Oklahoma	Savanna; Riding Horse	1521 [1989]	running walk [flat walk (slow); hard running walk (fast)]; rack [saddle rack (coon rack)]; broken pace [Indian shuffle]; pace	0.67 [0.71]	0.63	0.08	0.29	Promerová et al., 2014
Kentucky Natural Gaited	ca. 50	United	Kentucky	Riding Horse	ca. 1850	rack [saddle rack to	---	---	---	---	---

Horse [KN; see KS for location on map] [Kentucky Country Saddle Horse]		States			[2000]	hard rack]					
Kentucky Mountain Saddle Horse [KS] [Spotted Mountain Horse]	26,000	United States	Kentucky, Appalachians	Appalachian Mountains; Riding Horse	ca. 1850<br><br>[1989; 2002]	rack [mountain pleasure rack, saddle rack to hard rack]<br><br>[some]<br><br>running walk [flat walk (slow); hard running walk (fast)]<br><br>[some]<br><br>broken trot [fox trot]	0.92<br><br>[1.00]<br><br>0.94<br><br>[1.00]	0.84<br><br>0.88	0.16<br><br>0.12	0.00<br><br>0.00	Promerová et al., 2014<br><br>Gaspar et al., 2021
McCurdy Plantation Horse [MP] [McCurdy Walker]	750	United States	Alabama, Dallas and Lowndes Counties	Riding Horse	ca. 1890	running walk [flat walk (slow); hard running walk (fast)]; rack [saddle rack to hard rack (McCurdy lick or plantation gait	---	---	---	---	---

						or saddle gait)]; broken pace					
Missouri Fox Trotter [FT] [American Fox Trotter]	100,000	United States	Missouri, Ozarks	Ozark Mountains ; Riding Horse	1821<br><br>[1948 ; 1986]	broken trot [fox walk or flatfoot walk or flat walk (slow); fox trot (fast)]	1.00<br><br>[1.00]	1.00	0.00	0.00	Promerová et al., 2014; Gaspar et al., 2021
Montana Travler [TR]	300	United States	Montana	Mountains; Riding Horse	1973<br><br>[1979]	running walk [flat walk (slow); hard running walk (fast)]	---	---	---	---	---
Morgan [MO] [Gaited Morgan]	45,000	United States	Massachusetts, Springfield; Vermont	Riding Horse;	1789	[some]<br><br>running walk [flat walk (slow); hard running walk (fast)]; rack [saddle rack (pleasure gait) to hard rack]; broken pace; pace; broken trot [fox walk, park walk (slow) to fox trot, park	0.14<br><br>[0.26]<br><br>0.24<br><br>[0.42]<br><br>0.35<br><br>[0.51]	0.02<br><br>0.05<br><br>0.18	0.24<br><br>0.37<br><br>0.33	0.74<br><br>0.58<br><br>0.49	Promerová et al., 2014;<br><br>Jäderkvist Fegraeus et al., 2014;<br><br>Gaspar et al., 2021

						trot (fast)]					
Mountain Pleasure Horse [OK; see RM for location] [Old Kentucky Saddler; Kentucky Mountain Horse; Kentucky Saddle Horse]	ca. 500	United States	Kentucky, Appalachians	Appalachian Mountains; Riding Horse	ca. 1820<br><br>[1989]	rack [saddle rack (pleasure rack, trail rack)]	---	---	---	---	---
Narragansett Pacer [NP]	0	United States	Rhode Island, Narragansette Bay	Riding Horse; Racing Horse	1724 - 1880<br><br>[Extinct]	rack; broken pace; pace	---	---	---	---	---
National Show Horse [NS]<br><br>[Cross of Arabian + Saddlebred]	ca. 5000	United States	Arizona, Kentucky, Ohio, Dayton	Show Horse	[1981]	[some]<br><br>rack [saddle rack to speed rack]; broken pace [slow gait]	---	---	---	---	---
National Spotted Saddle Horse [SS]	ca. 25,000	United States	Tennessee, Central Region	Riding Horse	ca. 1970<br><br>[1979; 1985]	running walk [flat walk (slow); hard running walk (fast)]; rack [saddle rack to hard rack]; broken pace [not ideal]; broken trot [fox trot]	1.00<br><br>[1.00]	1.00	0.00	0.00	Gaspar et al., 2021

Newfoundland Pony [NF]	400	Canada	Newfoundland	Riding Horse; Working Horse	1612	Newfoundland Pony	Canada	Newfoundland	Riding Horse; Working Horse	1612	Newfoundland Pony
Nokota [NK] [Cow Pony]	500	United States	North Dakota, Theodore Roosevelt National Park	Feral	ca. 1830; 1850 [1989]	[some] broken pace [Indian shuffle]; broken trot	0.00 [0.00]	0.00	0.00	1.00	Gaspar et al., 2021
North American Curly [NC] [American Bashkir Curly] [crossbred with Appaloosa, Missouri Fox Trotter, and Tennessee Walker]	5000	United States	Nevada, Lander County	Peter Hanson Mountains; Riding Horse	ca. 1820; 1898 [1971]	[some] running walk [flat walk (slow); hard running walk (fast)]; saddle rack [curly rack]; broken pace [curly shuffle; Indian shuffle]; broken trot [fox walk to fox trot]	0.17 [0.33] 0.70 [0.85] 0.26 [0.36]	0.00 0.55 0.17	0.33 0.30 0.19	0.67 0.15 0.64	Promerová et al., 2014; Jäderkvist Fegraeus et al., 2014 Gaspar et al., 2021
North American Single-Footing [SF] [North American Singlefooter]	750	United States	Georgia, Alabama	Riding Horse	[1981; 1991]	rack [saddle rack (country or trail rack) to hard rack (road gait; speed	---	---	---	---	---

						rack; singlefoot)]					
Paint [PT] [American Paint Horse]	475,000	United States	Great Plains; Rocky Mountains; Oklahoma; Texas	Show Horse; Riding Horse	[1965]	[some]<br><br>broken pace [Indian shuffle]	0.08<br><br>[0.16]<br><br>0.06<br><br>[0.12]	0.00<br><br>0.00	0.16<br><br>0.12	0.84<br><br>0.88	Promerová et al., 2014<br><br>Gaspar et al., 2021
Racking Horse [RH] [Racker]	ca. 50,000	United States	Alabama, Decatur	Riding Horse	[1981]	rack [hard rack (style rack; speed rack; singlefoot)]	---	---	---	---	---
Rocky Mountain Horse [RM] [Mountain Pleasure Horse]	25,000	United States	Kentucky, Appalachians	Appalachian Mountains; Riding Horse	1890<br><br>[1986; 1989]	rack<br><br>[saddle rack (show gait, trail rack, mountain rack); hard rack (pleasure gait)]	1.00<br><br>[1.00]<br><br>0.92<br><br>[1.00]	1.00<br><br>0.84	0.00<br><br>0.16	0.00<br><br>0.00	Promerová et al., 2014;<br><br>Gaspar et al., 2021
Sable Island Horse [SI]	300	Canada	Nova Scotia, Sable Island	Sable Island; Feral	1760; 1801<br><br>[2008]	[some]<br><br>running walk [prance]; rack [saddle rack to hard rack]; broken pace [Indian shuffle]; pace	---	---	---	---	---
Sierra Tarahumara [ST]	ca. 100	Mexico	Chihuahua, Sierra Madre Occide	Sierra Tarahumara Mountains;	1567	[some]	Sierra Tarahumara [ST]	Mexico	Chihuahua, Sierra Madr	Sierra Tarahumara Mountains;	1567

			ntal States	Cart Horse					e Occidental States	Cart Horse	
Smokey Valley Horse [VS; same location as KS]	ca. 100	United States	Kentucky, Appalachian Mountains, Smokey Valley Farm	Appalachian Mountains; Riding Horse	[1979]	running walk [flat walk (slow); hard running walk (fast)]; rack [saddle rack (traveling gait) to hard rack]	---	---	---	---	---
Spanish Mustang [SM] [Cerbat; Challis; Sulphur]	1500	United States	Utah, Needles and Ogden, Home Mountains; Arizona, Kingman, Marble Canyon, Cerbat Mountains; Idaho, Challis	Feral; Cerbat Mountains; Home Mountains	ca. 1687 [1957; 1990]	[some] running walk [flat walk (slow); hard running walk (fast)]; rack [saddle rack to hard rack]; broken pace	10.0 [0.20] 0.15 [0.25]	0.00 0.03	0.20 0.22	0.80 0.75	Promerová et al., 2014; Gaspar et al., 2021
Spanish Colonial Horse [SC] [Baca-Chica; Belsky; Brislawn; Havapai; Huasteca [Nahua]; Holbrook; Jones; Kiger; Mt. Taylor;	85,000	United States	Pryor Mountains, Montana; Little Bookcliffs, Utah; Kiger Gorge, Steens Mountain, Oregon; Belen, Cebolla	Feral; Riding Horse	ca. 1683; 1890; 1930 [1990; 1998; 2001; 2013]	[some] running walk [flat walk (slow); hard running walk (fast)]; rack [saddle rack to hard rack];	---	---	---	---	---

Pryor; Romero/ McKinley; Santa Cruz; Wilbur-Cruce; Wild Mustangs; Yates]			, and Mount Taylor, New Mexico; Santa Cruz Island, California; Huasteca, Veracruz; Arivaca and Grand Canyon, Arizona; Lubbock and Marathon, Texas; Sand Hills, Nebraska			broken pace; pace					
Standardbred [SA]	300,000	United States (also Sweden; Norway)	New York	Harness Racing	ca. 1849 [1879]	[some] pace	1.00 [1.00] 0.65 [0.90]	1.00 0.40	0.00 0.50	0.00 0.10	Promerová et al., 2014; Gaspar et al., 2021
Tennessee Walking Horse [TW]	425,000	United States	Tennessee	Rocky Bluegrass Hills; Plantations	ca. 1790 [1900]	running walk [flat walk (slow; trail gait); hard running walk (fast; big lick; pleasure gait)]	1.00 [0.99-1.00]	0.99-1.00	0.00-0.01	0.00 0.01	Promerová et al., 2014; Staiger et al., 2016; Gaspar et al., 2021

						[some] rack [saddle rack to hard rack]; broken pace; pace [not ideal]; broken trot [fox trot]					
Tennuvian [TU] [Cross of Peruvian Paso + Tennessee Walking Horse]	100	United States	Tennessee; Parker, Colorado	Riding Horse	[1991]	running walk [soft prance]	---	---	---	----	---
Tiger Horse [TH] [Caballo Tigre; Soulon]	125	United States	Pacific Northwest	Riding Horse	1938 [1994; 2011]	running walk [flat walk (slow); hard running walk (fast)]; broken pace [glider gait, Indian shuffle, amble]; broken trot	---	---	---	---	---
Trottingbred [TB]	ca. 2000	United States	New York; New England	Harness Racing	1960 [1977]	[some] pace	---	---	---	---	---
Utah Walkony [UW] [Cross of Tennessee Walker +	ca. 25	United States	Utah, Cache County, Logan	Riding Horse; Handicapped Riders	ca. 1990	running walk [flat walk (slow); hard running	---	---	---	---	---

Various Ponies]						walk (fast)]					
Virginia Highlander [VH] [Virginia Pocket Horse; Virginian Singlefooter; Virginia Speed Racker]<br><br>[Cross of Welsh Pony + Arabian, Tennessee Walker, Morgan, and American Saddlebred]	50	Virginia	Smyth County, Blue Ridge Mountains	Mountains; Riding Horse	1960<br><br>[1991]	rack [hard rack]	---	---	---	---	---
Walkaloosa [WL]<br><br>[Cross of Tennessee Walker (or Missouri Fox Trotter or Peruvian Paso + Appaloosa]	ca. 2000	United States	Oregon, California	Riding Horse	ca. 1710; 1938<br><br>[1983]	running walk [flat walk (slow); hard running walk (fast)]; rack; broken pace [Indian shuffle; Appaloosa shuffle]; pace [not ideal]; broken trot [fox trot]	0.80<br><br>[1.00]	0.60	0.40	0.00	Gaspar et al., 2021

The rack, broken pace, or pace also shows up on occasion in other horse breeds of North America including the 15,000 or so Azteca Horses [AZ] of Texcoco, Mexico, the 400 or so Banker Horses [Corolla] [BH] of the Shackleford Banks in North Carolina (which also may show a running walk) and the 300 or so Chincoteague Ponies [CT] of Assateague Island of Virginia in the United States (which sometimes pace), the around 1000 Morab Horses

of Clovis, California in the United States (which may also exhibit the broken trot), the 200 or so Galiceño [GC] of Yucatan, Mexico and Texas (which has also been said to possess a running walk), and possibly in the 7000 or so Canadian and 100 or so Lac La Croix [Ojibwe] Ponies of Canada.

Table S3: Demographics for Central and South American Gaited Horses.

CENTRAL AND SOUTH AMERICA											
Breed	Number of Individuals	Country	Region	Zone	Year	Gait	A-allele [AA+CA geno-types]	AA geno-type	CA geno-type	CC geno-type	Reference
Abaco Barb [AB] [Spanish Barb]	0	Bahamas	Great Abaco Island	Feral; Abaco Island	ca. 1800 - 2015<br>[Extinct]	[some]<br>running walk; broken pace; pace	---	---	---	---	---
Andean [AN] [Andino; Morochuco; Peruvian Criollo]	ca. 200	Peru	Ayacucho Department, Cangallo Province	Andes Mountains; Riding Horse	1531	running walk [paso llano]; broken pace [sobreandando]; pace [huachano, not ideal]; broken trot [pasitrote, not ideal]	---	---	---	---	---
Campeiro [CO] [Marchador das Araucárias]	ca. 500	Brazil	Santa Catarina State, Concórdia, Curitibanos, and Lages; Caxias do Sul	Mountains and Araucária Moist Forest; Riding Horse	1620<br>[1912; 1976]	rack [marcha de centro]; broken pace [marcha picada]; broken trot [marcha batida]; pace [andadura, not ideal]	---	---	---	---	---
Campolina [CA] [Campolino]<br>[Cross of Mangalarga Marchador and Saddlebred]	85,000	Brazil	Minas Gerais State, Entre Rios and Passa Tempo	Mountains and Hills; Riding Horse	1870<br>[1938]	rack [marcha de centro]; broken pace [marcha picada]; broken trot [marcha	0.89<br>[0.90]<br>[1.00]<br>[1.00]	0.79<br><br>0.29<br>0.44	0.11<br><br>0.71<br>0.56	0.10<br><br>0.00<br>0.00	Promerová et al., 2014<br>Manso Filho et al., 2015<br>m. batida

			Municipalities			batida]; pace [andadura, not ideal]	[1.00]<br>0.50<br>[1.00]	0.12<br>0.00	0.88<br>1.00	0.00<br>0.00	m. picada<br>Gaspar et al., 2021
Colombian Paso Fino [PC] [Colombian Criollo; Colombian Walking]	60,000	Columbia	Cundinamarca, Antioquia, Quindío, Caldas, Cauca	Riding Horse	1524<br>[1947]	rack [paso fino, classic fino, fino clásico, show gait, saddlerack]; pace (not ideal, andadura saltada]	0.94<br>[0.95]<br>1.00<br>[1.00]	0.94<br>1.00	0.01<br>0.00	0.05<br>0.00	Promerová et al., 2014<br>Novoa Bravo et al., 2018
Colombian Trocha Pura [TC]	100,000	Columbia	Cundinamarca, Antioquia, Quindío, Caldas, Cauca	Andes Mountains; Riding Horse	1524<br>[1947]	[some]<br>rack [paso fino, saddlerack]<br>broken trot [trocha]	0.10<br>[0.18]<br>0.01<br>[0.02]	0.03<br>0.00	0.15<br>0.02	0.82<br>0.98	Promerová et al., 2014<br>Novoa Bravo et al., 2018
Colombian Trocha y Galope [TG; same location as TC]	1000	Columbia	Cundinamarca, Antioquia, Quindío, Caldas, Cauca	Andes Mountains; Riding Horse	1524<br>[1947]	[some]<br>broken trot [trocha]	0.25<br>[0.50]<br>0.04<br>[0.07]	0.00<br>0.00	0.50<br>0.07	0.50<br>0.93	Promerová et al., 2014<br>Novoa Bravo et al., 2018
Cuban Paso [CU] [Cuban Criollo; Cubano de Paso]	ca. 800	Cuba	Granma and Pinar, Provinces	Riding Horse	1511<br>[1961]	rack [marcha fina y gualdrapeada; paso del gualdrapeo]	---	---	---	---	---
Mangalarga Marchador [MM] [Sublime]	600,000	Brazil	Rio de Janeiro State, Paty do Alferes and Sul de Minas	Tropical Forest; Riding Horse	1740<br>[1949]	rack [marcha de centro]; broken pace [marcha picada]; broken trot [marcha	0.46<br>[0.68]<br>m. batida	0.23<br>0.15<br>0.31	0.45<br>0.00<br>0.65	0.32<br>0.85<br>0.04	Promerová et al., 2014<br>Manso Filhlo et al., 2015

			Municipalities; Minas Gerais State			batida]; pace [andadura, not ideal]	m. picada<br>[0.49]<br>m. batida<br>m. picada<br>[0.40]<br>m. batida<br>m. picada<br>m. batida<br>m. picada<br>0.49<br>[0.71]	0.26<br>0.00<br>0.57<br>0.20<br>0.00<br>0.52<br>0.06<br>0.87<br>0.27	0.23<br>0.07<br>0.43<br>0.20<br>0.06<br>0.43<br>0.34<br>0.13<br>0.44	0.21<br>0.93<br>0.00<br>0.60<br>0,94<br>0.05<br>0.60<br>0.00<br>0.29	Patterson et al., 2015<br>Fonseca et al., 2017<br>Bussiman et al., 2019<br>Gaspar et al., 2021
Mangalarga Paulista [Brazilian Saddle Horse; Mangalarga; Sela Brasileiro]	ca. 100,000	Brazil	São Paulo	Riding Horse	1740	[some]<br>broken pace [marcha picada]; broken trot [marcha batida]; pace [andadura, passo esquipado]	0.07<br>[0.14]<br>0.11<br>[0.22]	0.00<br>0.00	0.14<br>0.22	0.86<br>0.88	Promerová et al., 2014<br>Gaspar et al., 2021
Mangolina [MA] [cross of Mangalarga Marchador + Campolina]	ca. 1000	Brazil	Minas Gerais State	Mountains and Hills; Riding Horse	ca. 1993	rack [marcha de centro]; broken pace [marcha picada]; broken trot [marcha	---	---	---	---	---

						batida]; pace [andadura, passo esquipado, not ideal]					
Nordestino [ND] [Crioulo Nordestino; Mourão; Pé Duro; Sertanejo do Nordeste]	ca. 200,000	Brazil	Sertão, Bahia, Ceará, Paraíba, Pernambuco, Piauí, and Rio Grande do Norte States	Semiarid Desert and Scrubland; Cart Horse; Riding Horse	1549<br>[1987-2017]	[some]<br>broken pace [marcha picada]; broken trot [marcha batida]; pace [andadura, not ideal]	---	---	---	---	---
Pampa [PM] [Brazilian Criollo; Brazilian Crioulo]	50,000	Brazil	Rio Grande do Sul State	Pampas Grasslands and Moist Forest; Riding Horse	1536<br>[1980]	[some]<br>broken pace [marcha picada]; broken trot [marcha batida; trocha]; pace [andadura, not ideal]	0.02<br>[0.05]<br>0.00<br>[0.00	0.00<br>0.00	0.05<br>0.00	0.95<br>1.00	Promerová et al., 2014<br>Gaspar et al., 2021
Paso Fino [PR] (Puerto Rican Paso Fino; Pura Raza Paso Fino)	3000	Puerto Rico, Isla de Vieques; United States	Widespread	Mountains; Riding Horse	1511<br>[1943; 1964; 1966]	rack [paso fino, classic fino, fino clásico, or show gait (slow), paso corto, pleasure gait or trail gait (some, medium), paso largo (some, fast)]; broken trot [paso corto, pleasure gait or trail gait, most, medium], paso largo, most, fast];	0.99<br>[1.00]<br>0.97<br>[1.00]<br>[1.00]	0.99<br>0.95<br>1.00	0.01<br>0.05<br>0.00	0.00<br>0.00<br>0.00	Promerová et al., 2014;<br>Gaspar et al., 2021;<br>Wolbsberger et al., 2022

						broken pace [sobre paso, not ideal]; pace [andadura, not ideal]					
Paso Higueyano [PH]<br><br>[Cross of Criollo + Tennessee Walking Horse and Morgan]	ca. 500	Dominican Republic	Widespread	Riding Horse	1508; 1916<br><br>[2002]	stepping pace [paso higueyano]; pace; fox trot [some, paso higueyano]	---	---	---	---	---
Peruvian Paso [PP] [Costeño; Costeño de Paso Aclimatado a la Altura; Peruvian National Horse; Peruvian Stepping Horse]	35,000	Peru; United States	Cajamarca, Callejon de Huaylas, Huancayo	Coastal Mountains; Riding Horse	1531<br><br>[1992]	running walk [paso llano, ambladura rota]; broken pace [sobreandando (fast) aguilillo (slow), not ideal)]; pace [huachano, paso portante, ambladura (fast), entrepaso (medium), neither ideal]; broken trot [pasitrote, not ideal]	1.00<br><br>[1.00]<br><br>0.83<br><br>[1.00]	1.00<br><br><br><br>0.67	0.00<br><br><br><br>0.33	0.00<br><br><br><br>0.00	Promerová et al., 2014<br><br>Gaspar et al., 2021
Piquira [PQ] [cross between Brazilian Criollo and Shetland Ponies]	ca. 500	Brazil, Bahia State	Widespread	Riding Horse	1970	broken pace [marcha picada]; broken trot [marcha batida]; pace [andadura, not ideal]	---	---	---	---	---
Puerto Rican Criollo [RC] [same	ca. 500	Puerto Rico	Widespread	Riding Horse	1511	rack [paso fino, classic fino, fino	0.87<br><br>[0.99]	0.76	0.23	0.01	Wolfsberger et al., 2022

location as PR]						clásico (slow); paso corto (some, medium), paso largo (some, fast); broken trot [paso corto, most, medium], paso largo [most, fast]; broken pace [sobre paso, not ideal]; pace [andadura, not ideal]					

Some of the 150,000 Marajoara Island Horses [MJ] of Brazil have broken paces or broken trots. Alternative gaits of the broken pace may also be found on occasion in the ca. 10,000 Bolivian Paso [Sunicho] of Altiplano, and perhaps in the 10,000 or so Costa Rican Saddle Horse. And the ca. 500 Venezuelan Criollo Horses [VC] [Llanero] of Apura, Aragua, and Merida States sometimes has a rack or broken pace gait. The 1500 or so Lavradeiro of Roraima State in Brazil may also be gaited.

Table S4: Demographics of European Gaited Breeds (and Central Africa).

EUROPE											
Breed	Number of Individuals	Country	Region	Zone	Year Founded	Gait(s)	A-allele [AA+CA genotypes]	AA genotype	CA genotype	CC genotype	Reference
Aegidienberger [AD]<br><br>[Cross of Icelandic + Peruvian Paso]	600	Germany	North Rhine-Westphalia, Aegidienberg	Riding Horse	1994	rack [tölt, saddle rack to hard rack]	---	---	---	---	---
Albanian [AL] [Mysekaja; Myzeqeja; Myzequea]	ca. 3000	Albania	Albanian Alps; Myzeqeja Lowlands; Shkodra	Mountains and Plains; Pack Horse; Harness Horse	ca. 1386; 1904<br><br>[1990]	[some]<br><br>broken pace	---	---	---	---	---

Arravani [AR] [Macedonian Pacer]	300	Greece	Western Macedonia	Mountains; Riding Horse	ca. 1460	broken pace [arravani]	0.99<br><br>[1.00]	0.98	0.02	0.00	Giantsis et al., 2018; Antikas, 2015
Bidet Breton [BB] [Bidet d'allure; Carrossier Normand; Roncin; Roussin]	0	France	Brittany; Normandy, Western Region	Armorican Massifs [Mountains]; Riding Horse	ca. 1096-ca. 1700<br><br>[Extinct]	broken pace; pace; broken trot [?]	---	---	---	---	---
Castilian [CS] [Caballo Castellano]	0	Spain	Castile	Meseta Central Plateau and Mountains; Riding Horse	Ca. 800 BC-1700 CE<br><br>[Extinct]	running walk [flat walk; hard running walk; paso castellano]	---	---	---	---	---
Celtic Asturcón [CT; same location as SJ] [Asturian; Haubini]	0	Spain	Asturia	Asturian Mountains; Riding Horse	ca. 800 BC-1300 CE<br><br>[Extinct]	rack; broken pace; pace	---	---	---	---	---
Cretan [CR] [Messara]	1000	Greece	Crete, Messara	Mountains and Plains; Riding Horse; Sport Horse	1646	broken pace [arravani]	0.98<br><br>[1.00]	0.95	0.05	0.00	Promerová et al., 2014; Antikas, 2015
Dongola [DN] [Bandiagara, Djerma; Dongolawa; Fulani; Ganston; Hauossa; Hausa; Kréda; Mossi; North Sudan	ca. 500	Cameroon, Far North; Chad, Bahr-el-Ghazal; Nigeria, Borno	Dongola	Semi-Arid Sahel Desert; Riding Horse	ca. 1250	[some]<br><br>broken pace [takama]; pace	0.00<br><br>[0.00]	0.00	0.00	0.00	Promerová et al., 2014 [North Sudan Pony]

Pony; Songhai; Yagha]											
Faeroe Island Pony [FI] [Faeroese]	100	Denmark	Faeroe Islands	Island	ca. 700	[some] rack [tölt, saddle rack to hard rack or singlefoot]; broken pace [not ideal]; pace	0.14 [0.29]	0.00	0.29	0.71	Promerová et al., 2014
Galician [GA] [Andadura Gallega; Galiciana; Gallego de Andadura; Pura Raza Gallega]	1700	Spain	Galicia	Galician Mountains; Feral; Riding Horse; Racing Horse	ca. 800 BC [1997]	broken pace [andadura chapeada; singlefoot]; pace [andadura serrada]	0.50 [0.66]	0.33	0.33	0.33	Promerová et al., 2014
Galloway Pony [GW]	0	England	Scotland, Galloway; Yorkshire, Swaledale	Scottish Highlands; Riding Horse	ca. 1600 - 1800 [Extinct]	rack	---	---	---	---	---
Garrano [GR] [Marrano; Minho]	2000	Portugal	Minho, Tràs-os-Montes, Peneda-Géres National Park	Mountains; Riding Horse; Racing Horse; Pack Horse	ca. 700 BC [2011]	rack [hard rack or passo travado]; broken pace; pace [andadura]	---	---	---	---	---
Hobby Pony [HB] [Hobin; Ubinus]	0	Ireland	Connacht, County Kildare	Bogs; Riding Horse	ca. 1250 - 1800	rack	---	---	---	---	---

					[Extinct]						
Icelandic [IC]	80,000	Iceland	Vesturland, Norðurland vestra, Suðurland	Mountainous Island; Riding Horse	874<br>[1959]	rack [tölt, saddle rack to hard rack]; broken pace [skeiðtölt, skeiðborið tölt, fjórtakta skeið, not ideal]; pace [flying pace; flugskeið]; broken trot [brokktölt, not ideal]	0.75<br>[0.95]<br>0.73<br>[0.93]	0.53<br>0.53	0.42<br>0.40	0.05<br>0.07	Promerová et al., 2014;<br>Gaspar et al., 2021;<br>Wutke et al,. 2016
Mérens [ME] [Ariègeois; Castillon; Mérengais; Méringais]	1200	France	Pyreneesand Midi-Pyrenees, Ariege	Ariègeois and Pyrenees Mountains; Feral; Riding Horse; Cart Horse	ca. 1250<br>[1933]	[some]<br>broken pace; pace	---	---	---	---	---
Palfrey [PF] [Haquenée; Palfredus; Palefroi]	0	England, France, Italy	Canterbury, Devonshire, Durham, Ellesmere; Auvergne and Loire; Padua and Pavia	Riding Horse; War Horse	ca. 1200 - 1600<br>[Extinct]	running walk [?]; rack; broken pace; pace	---	---	---	---	---

Peneia [PE] [Georgalídiko; Pinia; Ravani]	100	Greece	Peloponnesus, Elis	Mountains; Riding Horse	ca. 1940 [1995]	broken pace [arravani]	0.97 [1.00]	0.94	0.06	0.00	Promerová et al., 2014; Antikas, 2015
Pindos [PI]	3500	Greece	Epirus	Pindus Mountains	ca. 300 BC	[some] broken pace [arravani]	0.23 [0.40]	0.07	0.33	0.60	Promerová et al., 2014 ; Antikas, 2015
Rhodian [RO] [Rhodes]	10	Greece	Rhodes	Island	ca. 300 BC [2001]	[some] broken pace	0.42 [0.67]	0.17	0.50	0.33	Promerová et al., 2014
Spanish Jennet [SJ] [Genet; Genete; Ginecti]	0	Spain	Asturia	Riding Horse	ca. 800-1700 [Extinct]	running walk [?]; rack; broken pace; pace; broken trot [?]	---	---	---	---	---
Thessalian [TH]	600	Greece	Thessaly	Riding Horse	ca. 350 BC	stepping pace [?]	0.25 [0.40]	0.10	0.30	0.60	Promerová et al., 2014
Welsh Mountain Pony [WM] [Section A]	4000	England; United States	Wales	Cambria Mountains	ca. 1485 [1902]	[some] pace	0.09 [0.19]	0.00	0.19	0.81	Promerová et al., 2014
Welsh Pony [Same location as WM] [Merlins; Welsh Pony of Cob Type; Welsh Pony of Riding Type; Sections B and C]	2000	England; United States	Wales	Riding Horse	ca. 1485 [1902]	[some] pace	0.07 [0.15] 0.07 [0.13]	0.00 0.00	0.15 0.13	0.85 0.87	Promerová et al., 2014; Gaspar et al., 2021

York [same location as PF]	0	England	York, Coppergate [Jorvik]	Riding Horse	750-800<br>[Extinct]	[?]<br>likely rack; broken pace; pace	1.00<br>[1.00]	1.00	0.00	0.00	Wutke et al., 2016
HARNESS RACERS											
Bulgarian Trotter [BT]	600	Bulgaria	Pazardzhik, Pleven, St. Zagora	Harness Racing	1962	[some]<br>pace	0.98<br>[1.00]	0.96	0.04	0.00	Lukanova et al., 2021
Dutch Harness [DH] [Tuigpaard]	2100	Netherlands	Friesland, Groningen	Harness Racing	1945<br>[1969]	[some]<br>pace	0.25<br>[0.25]	0.25	0.00	0.75	Gaspar et al., 2021
Finnhorse [FH] [Finnish Horse; Finnish Universal]	19000	Finland	Espoo, Kouvola, Lahti, Mikkeli, Turku	Harness Racing	ca. 1500<br>[1907]	[some]<br>pace	0.63<br>[0.89]<br>0.54<br>[0.87]	0.37<br>0.20	0.52<br>0.67	0.11<br>0.14	Jäderkvist Fegraeus et al., 2015;<br>Kvist et al., 2021
French Trotter [NT] [Norman Trotter]	20000	France	Normandy	Harness Racing	1836<br>[1922]	[some]<br>pace	0.78<br>[0.95]<br>0.17<br>[0.33]<br>0.80<br>[1.00]<br>0.77<br>[0.96]	0.61<br>0.00<br>0.60<br>0.59	0.34<br>0.33<br>0.40<br>0.37	0.05<br>0.67<br>0.00<br>0.04	Promerová et al., 2014 ;<br>Gaspar et al., 2021<br>Lukanova et al., 2021<br>Ricard et al., 2021 ; Khrabrova et al., 2020

German Trotter [GT]	2800	Germany	Gelsenkirchen	Harness Racing	ca. 1875	[some] pace	0.56 [0.66]<br>1.00 [1.00]	0.44<br>1.00	0.22<br>0.00	0.34<br>0.00	Promerová et al., 2014<br>Lukanova et al., 2021
Italian Trotter [IT] [Trottatore Italiano]	3000	Italy	Padova, Ferrara	Harness Racing	1862	[some] pace	0.87 [0.97]	0.76	0.21	0.02	Dall'Olio et al., 2021
Spanish Trotter [Trotador Español]	16000	Spain	Balleariс Islands, Mallorca and Menorca	Harness Racing	ca. 1890 [1970]	[some] pace	0.87 [0.97]	0.76	0.21	0.03	Negro Rama et al., 2016
Swedish Standardbred (Swedish Trotter)	16000	Sweden; United States	Boden, Gothenburg, Malmö. Mantorp, Stockholm	Harness Racing	ca. 1900	[some] pace	0.94 [0.97]<br>1.00 [1.00]	0.94<br>1.00	0.06<br>0.00	0.00<br>0.00	Promerová et al., 2014<br>Lukanova et al., 2021
Nordic Trotter [NT] [Norsk Kaldblodstraver; Svensk Kallblodstravare; Scandanavian Coldblood Trotter; Swedish-Norwegian Coldblooded Trotter]	8000	Denmark; Norway; Sweden	Copenhagen; Bergen, Drammen, Eidsberg, Oslo, Stavanger, Tønsberg; Trondheim; Boden, Gothenburg, Malmö. Mantorp, Stockholm	Harness Racing	1924 ; 1939<br>[1964]	[some] pace	0.45 [0.74]<br>0.35 [0.60]	0.16<br>0.09	0.58<br>0.51	0.26<br>0.40	Promerová et al., 2014;<br>Jäderkvist Fegraeus et al., 2017

A rack is also occasionally found in the 300 Alter Real [RE] of Alter do Chão, Portugal, and the stepping pace or pace in a few of the more than 170,000 Andalusian [Pura Raza Española] and 2500 modern Asturcón of Spain [same location as SJ], the million Arabians, the 9000 Gotlands of Sweden, the 3000 Lewitzers of Germany, and possibly the 100 or so Retuertas and 250 or so Pottoks of Spain and the 10,000 or so Bosnian Mountain horses. A pace also occurs in the Andalusian donkey.

Table S5: Demographics for Asian and South African Gaited Horses.

ASIA AND SOUTH AFRICA											
Breed	Number of Individuals	Country	Region	Zone	Year Founded	Gait(s)	A-allele [AA+CA genotypes]	AA genotype	CA genotype	CC genotype	Reference
Akhal-Teke	7000	Turkmenistan; United States	Ahal Region, Gökdepe District	Karakum Desert; Riding Horse	ca. 1000	[some] broken trot [glide]	0.00 [0.00]	0.00	0.00	0.00	Prome rová et al., 2014
Anadolu Yerli [AY] [Anatolian Native; Turkish Pony]	950,000	Turkey	Eastern Anatolia, Ağrı, Kars, and Van Provinces	Anti-Taurus Mountains and Steppes; Riding Horse; Racing Horse; Harness Horse; Sport Horse	ca. 1000	broken pace [yorga]; pace [rahvan]	0.91 [0.96]	0.86	0.10	0.04	Özbeyaz, et al., 2016
Buryat [BY] [Buryatskaya; Byryatskaya]	10000	Russia	Siberia, Buryatia Republic	Baikal Mountains and Taiga; Riding Horse	ca. 1207	[some] broken trot [tropota]	---	---	---	---	---
Canik [CN]	5000	Turkey	Amasya, Samsun, and Tokat Provinces	Pontic Mountains; Riding Horse	ca. 1000; 1800	broken pace [yorga]; pace [rahvan]	---	---	---	---	---
Chakouyi [CK]	10000	China	Gansu Province, Lanzho	Gobi Desert; Qilian Mounta	ca. 100 BC	pace	0.96 [1.00]	0.92	0.08	0.00	Han et al., 2015

			u, Tianzhu, Wuwei Districts	ins; Riding Horse; Racing Horse			0.82<br>[0.96]	0.67	0.29	0.04	Li et al., 2022
Cheju [Jeju] [CH]	2100	South Korea	Jeju Province	Mountainous Island; Riding Horse; Racing Horse	1276	[some]<br>pace	0.14<br>[0.29]<br>0.50<br>[0.75]	0.00<br>0.25	0.29<br>0.50	0.71<br>0.25	Prome rová et al., 2014;<br>Srikanth et al., 2020
Datong [DT]	23000	China	Qinghai Province, Datong River Basin	Riding Horse	ca. 100 BC	pace	0.54<br>[0.85]	0.22	0.63	0.15	Han et al., 2015
Ferghana [FG] [Dayuan; Turkoman]	0	Uzbekistan	Fergana Valley	Riding Horse	ca. 104 BC-1000 CE<br>[Extinct]	broken pace; pace	---	---	---	---	---
Gansu [Heavenly Horse ; Soulon; Tiger Horse; Wusun]	0	China	Gansu Province, Wuwei; Sichuan Province	Riding Horse	618-1000<br>[Extinct]	broken pace; pace	---	---	---	---	---
Hokkaido [HK] [Dosanko]	2000	Japan	Hokkaido Island	Mountainous Island; Riding Horse; Racing Horse	ca. 1600<br>[1979]	[some]<br>pace	0.72<br>[0.85]<br>0.18<br>[0.34]<br>pacers	0.58<br>0.02<br>1.00	0.27<br>0.32<br>0.00	0.15<br>0.66<br>0.00	Prome rová et al., 2014;<br>Chandra et al., 2020<br>Amano et al., 2018
Kabarda [KD] [Kabardian	1000	Russia; Georgia	Stavropol Krai,	Caucasus Mounta	ca. 1500	[some]	0.00<br>[0.00	0.00	0.00	1.00	Prome rová

; Kabardin; Kabardinskaya]			Kabardino-Balkaria Republic	ins and Steppes; Riding Horse		broken trot [tropota]; broken pace; pace					et al., 2014
Kalmyk [KM] [Astrakhan; Kalmutskaya; Kalmyskaya]	2000	Russia	Volga River Basin, Astrakhan and Volgograd	Steppes; Riding Horse	ca. 1635	[some] broken pace; pace	---	---	---	---	---
Karachai [KC] [Karachayevskaya; Karachay; Karachey]	20000	Russia	North Caucasus, Karachay-Cherkessia Republic	Caucasus Mountains; Riding Horse	ca. 1500 [1935]	[some] broken trot [tropota]; broken pace; pace	---	---	---	---	---
Kathiawari [KW] [Kathi; Kutchi]	8000	India	Gujarat, Kachchh, Kathiawar, and Surat Districts	Thar Desert; Riding Horse	ca. 1526	[some] broken trot [revaal rewal, aphcal]	---	---	---	---	---
Kazakh [KZ] [Adaev; Deliboz; Delibozskaya; Dilbaz; Diliboz; Dzhab; Gazakh; Jabe; Kazakhskaya; Qazaq; Wusun]	300000	Kazakhstan; China	Central Aktobe Region, Aghstafa, Qazax, Shaki, Tovuz, and Zagastala Districts	Steppes; Riding Horse	ca. 450 BC; 1800 [1943]	[some] broken pace; pace	0.38 [0.63]	0.13	0.50	0.37	Han et al., 2015
Kyrgyz [KG] [Kirgis; Kirgiz;	80000	Kirghizstan	Widespread	Tian Shan Mountains; Riding	ca. 201 BC	[some] broken	0.40 [0.58]	0.23	0.35	0.42	Promerová et al., 2014;

Kirgizskaya]				Horse; Racing Horse; Sports Horse		pace; pace	0.46	---	---	---	Kalinkova et al., 2020
Marwari [MW] [Malani]	3000	India	Marwar, Rajasthan	Thar Desert; Riding Horse; Show Horse	ca. 1193; 1526	broken trot [revaal, rewal, aphcal]	0.00 [0.00]<br>0.15	0.00<br>---	0.00<br>---- [some in Jun, Staiger]	0.00<br>----	Promerová et al., 2014<br>Staiger et al., 2017; Jun et al., 2014
Mongolian [WU] [Chinese Mongolian [Ujumqin; Wushen]; Mongolian [Darkhad; Galshar; Tes]	20000	China; Mongolia	Inner Mongolia; Khövsgöl, and Töv Provinces	Gobi and Maowusu Deserts; Steppes; Khangai and Khentii Mountain; Riding Horse; Racing Horse	ca. 600	[some] broken pace; pace [joroo]	0.05 [0.07]	0.01	0.06	0.93	Promerová et al., 2014; Kalinkova et al., 2022; Han et al., 2023;
Mytilene [MI] [Ayvacik Midillisi; Ege Midillisi; Lesvos]	300	Turkey	Anatolia, Canakkale Province, Midillisi Island; Lesvos Island, Greece	Island Horse; Riding Horse	ca. 1299	broken pace [yorga]; pace [rahvan]	---	---	---	---	---
Nisean [same location as TM]	0	Iran, [Bactria]	Hamadan Province	Nisean Plains; Zagros Mountains; Riding Horse	522 BC-1204 CE	broken pace; pace [Extinct]	---	---	---	---	---

Orlov Trotter [OT]	1200	Russia	Voronezh Governorate	Harness Racing	ca. 1800	[some] pace	0.20 [0.40]	0.00	0.40	0.60	Promovera et al., 2014; Kalinkova et al., 2022
Russian Trotter [RT]	300000	Russia	Orel Region, Bolkhov District	Harness Racing	1949	[some] pace	---	---	---	---	---
Shan [SH] [Burmese; Burmin; Burman; Kachin]	25000	Myanmar	Shan	Shan Mountains; Riding Horse; Sport Horse	ca. 950	[some] broken pace [ahthacha]; pace	0.02 [0.05]	0.00	0.05	0.95	Okuda et al., 2017
Sindhi [SI] [Kachchhi-Sindhi; Makra; Samoundi, Sindhi-Katchi; Sindhi-Kathiawari]	100	Pakistan; India	Sindh Province, Rajasthan, Jaisalmer-Barmer District	Thar Desert; Riding Horse; Racing Horse	ca. 1526	broken trot [revaal, rewal, aphcal]	---	---	---	---	---
Spiti [SI] [Chamurthi; Chamurti; Chummarti]	1000	India	Himachal Pradesh, Kangra, Kinnaur, Kullu, Mandi, and Shimla Districts, Kangra, Pin, and Spiti Valleys; Ladach,	Himalayan Mountains; Riding Horse	ca. 618	broken pace; pace	---	---	---	---	---

			Changtha Plateau; Pin Valley, Lahoul								
Tibetan [TI] [Ganzi; Maiwa; Nangchen; Rdochurta; Tibetanan-Sikang Pony; Yushu; Zhongdian]	300000	China	Qinghai (Yushu), Sichuan (Aba Tibet), Tibet (Chamdo)	Himalayan Mountains	ca. 800	broken pace; pace	0.39 [0.75]	0.04	0.71	0.25	Han et al., 2015
Transbaikal [TL] [Zabaikal; Zabaykalskaya]	300	Russia	Siberia, Transbaikal, Zabaykalsky Krai (Dauria)	Baikal Mountains; Taiga; Riding Horse	ca. 1300	broken trot [tropota]	0.00 [0.00]	0.00	0.00	0.00	Khrabrova et al., 2020
Turkmene [TK] [Turkmen; Turkmenian]	0	Turkmenistan; Iran	Karakum Desert	Karakum Desert; Riding Horse	ca. 700-1826 [Extinct]	broken pace; pace	---	---	---	---	---
Tushin [TN] (Tushiri Cxeni; Tushetian)	1200	Georgia	Tusheti	Caucasus Mountains; Riding Horse	ca. 300 BC	[some] broken pace; pace	0.47 [0.67]	0.27	0.40	0.33	Prome rová et al., 2014
Tuva [TV] [Tuvinskaya]	80,000	Russia	Tuva	Sayan and Tannu-Ola Mountains; Riding Horse; Harness Horse	ca. 1200. 1830 [1951]	[some] pace	0.17 [0.33]	0.00	0.33	0.67	Khrabrova et al., 2020
West Black Sea [WB] [Chernomor; Chernomor	50000	Russia	Black Sea, Krasnodar Krai and	Riding Horse; Racing Horse	ca. 1790 [1921]	broken pace [yorga]; pace [rahvan]	---	---	---	---	---

eskaya; Nogai; Rahvan]			Rostov Oblast Districts								
Yanqi [YQ] [Yanji]	20000	China	Bayingolin Mongol, and Xinjiang Regions, Bohu, Hejing, Hoxud, and Yanqi Hui Counties	Steppes; Riding Horse	ca. 1200; 1800	pace	0.33 [0.44]	0.13	0.41	0.46	Han et al., 2015
Yili [YL]	120000	China	Ili Kazakh and Xinjiang	Steppes; Riding Horse	1956	pace	0.39 [0.71]	0.06	0.65	0.29	He et al., 2017
Yakut [YK] [Yakutian; Yakutskaya]	102000	Russia	Siberia, Sakha Republic (Yakutia)	Mountains; Taiga; Riding Horse	ca. 1300	[some] broken trot [tropota]	0.00 [0.00]<br>0.04 [0.09]	0.00<br>0.00	0.00<br>0.09	1.00<br>0.91	Khrabrova et al., 2020;<br>Kalinkova et al., 2022

SOUTH AFRICAN HORSES											
Breed	Number of Individuals	Country	Region	Zone	Year Founded	Gait(s)	A-allele [AA+CA genotypes]	AA genotype	CA genotype	CC genotype	Reference
Afrikan Saalperd [SP] [South African Saddlebred]	ca. 1500	South Africa	Colesberg	Show Horse; Riding Horse	1918 [1949]	[some] rack [saddle rack or trippel to hard rack]; broken pace [slow gait or	---	---	---	---	---

						kortgang]					
Basuto [BS] [Transkei]	100000	Lesotho ; South Africa	Transkei	Maloti Mountains	1652 [1977]	[some] rack [saddle rack or trippel] ; broken pace [kortgang]; pace [passgang, not ideal]	0.07 [0.13]	0.00	0.13	0.87	Promerová et al., 2014
Cape Boer [CB] [Boer Pony; Kaapse Boerperd]	2000	South Africa	Transvaal, Orange Free State, Sandveld Preserve	Riding Horse	1652 [1957]	[some] rack [saddle rack or trippel] ; broken pace [slow gait or kortgang]	0.15 [0.30]	0.00	0.30	0.70	Promerová et al., 2014
Nooitgedacht [NG] [Nooitgedacht Transkei]	1500	South Africa	Transvaal, Ermelo	Riding Horse	1652 [1952 ; 1967]	[some] rack [saddle rack or trippel] ; broken pace [strykstap]; pace [passgang; not ideal]	0.21 [0.36]	0.07	0.29	0.64	Promerová et al., 2014

An alternative gait shows up on occasion in a few other Asian breeds as well. The pace or broken pace is sometimes found in the ca. 1000 Yamud [YM] [Iomud] of Turkmenistan [Velayats of Balkan and Daşoguz], the 300 or so Vyatka [VY] in the Kirov Oblast of Russia, the 2500 or so Bhutia [BU] [Chyanta; Yuta] of Bhutan, Nepal, and Sikkim India, the 350 Zaniskari [ZA] of Zanskar Valley, Ladakh, and Jammu-Kashmir, India, the 700 Caspian [Elburz Mountains], 2700 Kurdi [KU] [Jaf] [Provinces of Kurdistan and Kermanshah], and 400 Turkoman [TM] of Iran near the Alborz Mountains and Steppes, the 150000 Karabair [Bukhara] [KR] of Tajikistan and Uzbekistan, the 200 Karabakh [KK] in the Karabakh Mountains of Azerbaijan. The pace seems to occur in a few of the 50 or so remaining Taishuh [Taisho] horses of Tsushima Island, Nagasaki Prefecture, Japan, or at least it did in the past. The

pace might also occur in the 3100 Altai [AT] of the Altai Mountains in Mongolia, the 4000 Mezen of Siberia, Russia, and the 14000 Kushum [KS] of the Ural Mountains in Kazakhstan, and the 400 Dareshuri of the Zagros Mountains in Iran. Some of the Indonesian and Polynesian horses have also been described as gaited, though the evidence for this is unclear, such as the 48000 Sandalwood [SW] and 20000 Timor [MR] of Indonesia [and perhaps the 50000 Sumba], though the extinct Java [JV] horse seems to have led to gaited South African horses. Some South Asian breeds carry the DMRT3 allele and it is also possible they may be able to pace though they are not known to do so, including the 6000 Balikun of Xinjiang (Barkol Kazakh), China, 13000 Cadamu [Chaidamu] of the Qaidam Basin, China, 130000 Hequ in the Gansu, Sechuan, and Qinghai Provinces, China. The 300 or so Kaimanawa [KA] in the Kaimanawa Mountains and ca. 200 Nati [Ngati] [NA] horses on the East Coast of North Island, New Zealand, seem to be able to fox trot on occasion, and perhaps also can perform the broken pace as some of the 10000 or so Tongan Singlefooters [TS] from Tonga likely do.

References for Table S1

Back, W.; Schamhardt, H.C.; Barneveld, A. Are kinematics of the walk related to the locomotion of a warmblood horse at the trot? *Vet. Q.* **1996**, *18*, 79–84, doi:10.1080/01652176.1996.9694699.

Back, W.; Schamhardt, H.C.; Barneveld, A. Kinematic comparison of the leading and trailing fore- and hindlimbs at the canter. *Equine Vet. J.* **2010**, *29*, 80–83, doi:10.1111/j.2042-3306.1997.tb05060.x.

Barrey, E.; Desliens, F.; Poirel, D.; Biau, S.; Lemaire, S.; Rivero, J.L.L; Langlois, B. Early evaluation of dressage ability in different breeds. *Equine Vet. J.* **2002**, *34*, 319–324, doi:10.1111/j.2042-3306.2002.tb05440.x.

Bekker, L. Riding the gaited horse. *SA Horseman* **2009**, *4*, 32–35, doi:10.10520/EJC14392.

Bertram, J.E.A.; Gutmann, A. Motions of the running horse and cheetah revisited: Fundamental mechanics of the transverse and rotary gallop. *J. R. Soc. Interface* **2009**, *6*, 549–559, doi:10.1098/rsif.2008.0328.

Biknevicius, A.R.; Mullineaux, D.R.; Clayton, H.M. Locomotor mechanics of the tölt in Icelandic horses. *Am. J. Vet. Res.* **2006**, *67*, 1505–1510, doi:10.2460/ajvr.67.9.1505.

Bobbert, M.F.; Gómez Álvarez, C.B.; Weeren, P.R.; Roepstorff, L.; Weishaupt, M.A. Validation of vertical ground reaction forces on individual limbs calculated from kinematics of horse locomotion. *J. Exp. Biol.* **2007**, *210*, 1885–1896, doi:10.1242/jeb.02774.

Boehart, S.; Marquis, H; Falaturi, P; Carstanjen, B. Influence of palmarly added weights on locomotor parameters of the tölt of Icelandic Horses and comparison with corresponding data of the flying pace. *Pferdeheilkunde* **2013**, 29, 628–632, doi:10.21836/PEM20130508.

Boehart, S.; Massarf, L.; Marquis, H.; Falaturp, P.; Gabriel, A.; Carstanjen, B. Development of locomotor parameters of the flying pace of Icelandic Horses after application of weights to the palmar aspect of the hoof. *Pferdeheilkunde* **2012**, *28*, 597–602, doi:10.21836/PEM20120511.

Bruns, U. Experience with strange gaits. *Iceland. Horse Soc. Gt. Br. Newslett.* **1991**, *16*, 12–16.

Buchner, H.H.F.; Obermüller, S.; Scheidl, M. Body centre of mass movement in the sound horse. *Vet J.* **2000**, *160*, 225–234, doi:10.1053/tvjl.2000.0507.

Cano, M.R.; Miró, F.; Vivo, J.; Galisteo, A.M. Comparative biokinematic study of young and adult Andalusian horses at the trot. *Zentralbl. Veterinarmed. A.* **1999**, *46*, 91–101, doi:10.1046/j.1439-0442.1999.00196.x.

Cano, M.R.; Vivo, J.; Miró, F.; Morales, J.L; Galisteo, A.M. Kinematic characteristics of Andalusian, Arabian and Anglo-Arabian horses: A comparative study. *Res. Vet. Sci.* **2001**, *71*, 147–153, doi:10.1053/rvsc.2001.0504.

Clayton, H.M. Comparison of the stride kinematics of the collected, medium, and extended walks in horses. *Am. J. Vet. Res.* **1995**, *56*, 849–852.

Clayton, H.M. Comparison of the stride kinematics of the collected, working, medium and extended trot in horses. *Equine Vet. J.* **1994**, *26*, 230–234, doi:10.1111/j.2042-3306.1994.tb04375.x.

Clayton, H.M. Singleton, W.; Lanovaz, J.L.; Prades, M. Pastern joint motion in trotting horses *AAEP Proc.* **2000**, *46*, 217–219.

Clayton, H.M. *The Dynamic Horse: A Biomechanical Guide to Equine Movement and Performance*; Sport Horse Publications: Mason, Michigan, USA, 2004; pp. 161–194; ISBN 978-09-7476-700-0.

Clayton, H.M.; Bradbury, J.W. Temporal characteristics of the fox trot, a symmetrical equine gait. *Appl. Anim. Behav. Sci.* **1994**, *42*, 153–159, doi:10.1016/0168-1591(94)00539-Q.

Clayton, H.M.; Hodson, E.; Lanovaz, J.L. The forelimb in walking horses: 2. Net joint moments and joint powers. *Equine Vet. J.* **2000**, *32*, 295–300, doi:10.2746/042516400777032174.

Clayton, H.M.; Hodson, E.; Lanovaz, J.L. The hindlimb in walking horses: 2. Net joint moments and joint powers. *Equine Vet. J.* **2001**, *33*, 44–48, doi:10.2746/042516401776767359.

Clayton, H.M.; Sha, D.; Stick, J.; Elvin, D. 3D kinematics of the equine metacarpophalangeal joint at walk and trot. *Vet. Comp. Orthop. Traumatol.* **2007**, *20*, 86–91, doi:10.1160/vcot-07-01-0011.

Clayton, H.M.; Sha, D.H. Head and body centre of mass movement in horses trotting on a circular path. *Equine Vet. J. 2006*, *36*, 462–467, doi:10.1111/j.2042-3306.2006.tb05588.x.

Crawford, W.H.; Leach, D.H. The effect of racetrack design on gait symmetry of the pacer. *Can. J. Comp. Med.* **1984**, *48*, 374–380.

Deuel, M.S.; Lawrence, L.M. Kinematics of the equine transverse gallop. *J. Equine Vet. Sci.* **1987**, *7*, 375–382, doi:10.1016/S0737-0806(87)80008-4.

Deuel, N.R.; Park, J.J. 1990. The gait patterns of Olympic dressage horses. *J. Appl. Biomech.* **1990**, *6*, 198–226, https://doi.org/10.1123/ijsb.6.2.198.

Drevemo, S.; Dalin, D.; Fredricson, I. 1980. Equine locomotion: 1. The analysis of linear and temporal stride characteristics of trotting standardbreds. *Equine Vet. J.* **1980a**, *12*, 60–65, doi:10.1111/j.2042-3306.1980.tb02310.x.

Drevemo, S.; Fredricson, I.; Dalin, G.; Björne, K. Equine locomotion: 2. The analysis of coordination between limbs of trotting Standardbreds. *Equine Vet. J.* **1980b**, *12*, 66–70, doi:10.1111/j.2042-3306.1980.tb02311.x.

Duarte, H.O.G.; Rosa, G.S.; Hussni, C.A. Aspectos da locomoção e bases gerais dos andamentos básicos naturais dos equinos. *Vet. e Zootec.* **2022**, *29*, e1–12, doi:10.35172/rvz.2022.v29.1012.

Dutto, D.J.; Hoyt, D.F.; Clayton, H.M.; Cogger, E.A.; Wickler, S.J. Joint work and power for both the forelimb and hindlimb during trotting in the horse. *J. Exp. Biol.* **2006**, *209*, 3990–3999, doi:10.1242/jeb.02471.

Feldmann, W.; Rostock, A.K. *Islandpferde Reitlehre*. Gestüt Aegidienberg: Bonn, Germany, 1988, pp. 249-306.

Galisteo, A.M.; Cano, M.R.; Morales, J.L.; Miró, F.; Vivo, J.; Agüera, E. Kinematics in horses at the trot before and after an induced forelimb supporting lameness. *Equine Vet. J.* **1997**, *23*, 97–101, doi:10.1111/j.2042-3306.1997.tb05064.x.

Galisteo, A.M.; Vivo, J.; Cano, M.R.; Morales, J.J.; Miró, F.; Arguera, E. Differences between breeds (Dutch Warmblood vs. Andalusian Purebred) in forelimb kinematics. *J. Equine Sci.* **1997**, *8*, 43–47. [trot; did] https://doi.org/10.1294/jes.8.43.

Galisteo, A.M.; Vivo, J.; Miró, F.; Morales, J.L.; Monterde, J.G.; Cano, M.R. Variaciones en el patrón biocinemático básico del paso de caballos de tres razas guiados de la mano. *Arch. Zoot.* **1998**, *48*, 327–335.

Goubaux, A.; Barrier, G. *The Exterior of the Horse*; J.B. Lippincott: Philadelphia, 1892; pp. 472-575; ISBN 978-13-7664-079-3.

Gunnarsson, V.; Stefánsdóttir, G.J.; Jansson, A.; Roepstorff, L. The effect of rider weight and additional weight in Icelandic horses in tölt: Part II, stride parameters. *Animal* **2017**, *11*, 1567–1572, doi:10.1017/S1751731117000568.

Harrison, S.M.; Whitton, R.C.; King, M.; Haussler, K.K.; Kawcak, C.E.; Stover, S.M.; Pandy, M.G. Forelimb muscle activity during equine locomotion. *J. Exp. Biol.* **2012**, *215*, 2980–2991, https://doi.org/10.1242/jeb.065441.

Hildebrand, M. Analysis of asymmetrical gaits. *J. Mammal.* **1977**, *58*, 131–156, doi:10.2307/1379571.

Hobbs, S.; Licka, T.; Polman, R. The difference in kinematics of horses walking, trotting and cantering on a flat and banked 10m circle. *Equine Vet. J.* **2011**, *43*, 686–694, doi:10.1111/j.2042-3306.2010.00334.x.

Hobbs, S.J.; Bertram, J.E.; Clayton, H.M. An exploration of the influence of diagonal dissociation and moderate changes in speed on locomotor parameters in trotting horses. *PeerJ* **2016**, *4*, e2190, doi:10.7717/peerj.2190.

Hodson, E.; Clayton, H.M; Lanovaz, J.L. The forelimb in walking horses: 1. Kinematics and ground reaction forces. *Equine Vet. J.* **2000a**, *32*, 287–94, doi:10.2746/042516400777032237.

Hodson, E.; Clayton, H.M; Lanovaz, J.L. The hindlimb in walking horses: 1. Kinematics and ground reaction forces. *Equine Vet. J.* **2000b**, *32*, 38–43, doi:10.2746/042516401776767485.

Holmström, M.; Fredricson, I.; Drevemo, S. Biokinematic analysis of the Swedish Warmblood riding horse at trot. *Equine Vet. J.* **1994**, *26*, 235–240, doi:10.1111/j.2042-3306.1994.tb04376.x.

Imus, B. *Heavenly Gaits: The Complete Guide to Gaited Riding Horses*; Breakthrough Publications: New York, New York, USA, 1995; ISBN 978-09-1432-782-0.

Jordan, R. *The Gait of the American Trotter and Pacer*. William R. Jenkins: New York, New York, USA, 1910.

Khumsap, S.; Clayton, H.; Lanovaz, J.L. Effect of walking velocity on ground reaction force variables in the hindlimb of normal horses. *Am. J. Vet. Res.* **2001**, *62*, 901–906, doi10.2460/ajvr.2001.62.901.

Leleu, C.; Cotrel, C.; Barrey, E. Effect of age on locomotion of Standardbred trotters in training, *Equine Comp. Exerc. Physiol.* **2004**, *1*, 107–117, doi:10.1079/ECEP200312.

Miró, F.; Vivo, J.; Cano, R.; Diz, A.; Galisteo, A.M. Walk and trot in the horse at driving: Kinematic adaptation of its natural gaits. *Anim. Res.* 2006, *55*, 603–613, doi:10.1051/animres:2006038.

Morales, J.L.; Manchado, M.; Vivo, J.; Galisteo, A.M.; Agüera, E.; Miró, F. Angular kinematic patterns of limbs in elite and riding horses at trot. *Equine Vet. J.* **1998**, *30*, 528–533, doi10.1111/j.2042-3306.1998.tb04529.x.

Morales, J.L.; Manchado, M.; Vivo, J.; Galisteo, A.M.; Agüera, E.; Miró, F. Angular kinematic patterns of limbs in elite and riding horses at trot. *Equine Vet. J.* **1998**, *30*, 528–533, doi:10.1111/j.2042-3306.1998.tb04529.x.

Morales-Acosta, L.; Ortiz-Prado, A.; Jacobo-Armendáriz, V.H.; González-Carbonel, R.A. Analysis and characterization of the normal gait phases of walking Warmblood horses as a tool for the diagnosis of lameness. *Pesq. Vet. Bras.* **2018**, *38*, 536–543, doi:10.1590/1678-5150-pvb-4496.

Nauwelaerts, S.; Zarski, L.; Aerts, P.; Clayton, H. Effects of acceleration on gait measures in three horse gaits. *J. Exp. Biol.* **2015**, *218*, 1453–1460, doi:10.1242/jeb.113225.

Nicodemus, M. Temporal variables of the pleasure and road trots of the Morgan English Pleasure Horse. *J. Equine Vet. Sci.* **2009**, *5*, 308–309, doi:10.1016/j.jevs.2009.04.014.

Nicodemus, M.C.; Clayton, H.M. Temporal variables of four-beat, stepping gaits of gaited horses. *Appl. Anim. Behav. Sci.* **2003**, *80*, 133–142, doi:10.1016/S0168-1591(02)00219-8.

Nicodemus, M.C.; Holt, K.M.; Clayton, H.M. Temporal variables of the park walk and park trot of the Morgan Horse. *J. Anim. Sci. Suppl.* **2001**, *79*, 210.

Nicodemus, M.C.; Holtz, H.M.; Swartz, K. Relationship between velocity and temporal variables of the flat shod running walk. Equine Vet. J. **2010**, *34*, 340–343, doi:10.1111/j.2042-3306.2002.tb05444.x.

Nicodemus, M.C.; Lanovaz, J.L.; Clayton, H.M. The effect of velocity on temporal variables of the equine walk. In *Conference on Equine Sports Medicine and Science: The Elite Show Jumper*; Lider, A., Ed.; Lensing Druck: Dortmund, Germany, 2000; p. 155.

Nicodemus, M.C.; Slater, K. Comparisons of the Arabian Hunter and Country English Pleasure trot temporal variables. *Proc. Equine Sci. Soc.* **2007**, *20*, 246–247.

Nicodemus, M.C.; Slater, K. Forelimb kinematics of the flat walk and fox trot of the Missouri Fox Trotter. *Comp. Exerc. Physiol.* **2009**, *6*, 149–156, doi:10.1017/S1755254010000048.

Östlund, V. *Limb Phasing Icelandic Horses*. Thesis. Swedish University of Agricultural Sciences: Uppsala, Sweden, 2011.

Pecha, A.; Rumpler, B.; Kotschwar, A.; Peham, C.; Licka, T. The influence of weighted heel boots on the duration and start of the stance phases of all four limbs in slow and fast tolt in the Icelandic Horse. *Pferdeheilkunde* **2011,** *27*, 686–694, doi:10.21836/PEM20110617.

Pfau, T.; Witte, T.H.; Wilson, A.M. Centre of mass movement and mechanical energy fluctuation during gallop locomotion in the Thoroughbred racehorse. *The Journal of Experimental Biology* **2006**, *209*, 3742–3757, doi:10.1242/jeb.02439.

Plumb, C.S. The pacer. In *Types and Breeds of Farm Animals*; Ginn: Boston, Massachusetts, USA, 1906; pp. 49–54.

Renders, E.; Vincelette, A. Laterally coordinated gaits in the modern horse (*Equus ferus caballus*). In *Animal Husbandry*; Kukovics, S., Ed.; IntechOpen: London, UK, 2022; pp. 125–156; ISBN 978-18-0355-126-5.

Reynisson, G. *Analysis of Movement in Pace and Tölt in the Icelandic Horse*. Thesis. Agricultural University of Iceland: Borgarbyggð, Iceland, 2017.

Rhodin, M.; Smit, I.H.; Persson-Sjodin, E.; Pfau, T.; Gunnarsson, V.; Björnsdóttir, S.; Zetterberg, E.; Clayton, H.M.; Hobbs, S.J.; Serra Bragança, F.; Hernlund, E. Timing of vertical head, withers and pelvis movements relative to the footfalls in different equine gaits and breeds. *Animals* **2022**, *12*, e3053, doi10.3390/ani12213053.

Roberson, P. *Validation of a Three-Dimensional Motion Capture System for Use in Identifying Characteristics of the Running Walk*. Thesis. University of Tennessee: Knoxville, Tennessee, USA, 2007.

Rooney, J.R.; Thompson, K.N.; Shapiro, R. A contribution to the study of velocity, stride length, and frequency in the horse. *J. Equine Vet. Sci.* **1991**, *11*, 208–209, doi:10.1016/S0737-0806(06)80978-0.

Serra Bragança, F.M.; Broomé, S.; Rhodin, M.; Björnsdóttir, S.; Gunnarsson, V.; Voskamp, J.P.; Persson-Sjodin, E.; Back, W.; Lindgren, G.; Novoa-Bravo, M.; Gmel, A.I.; Roepstorff, C.; Van der Zwaag, B.J.; Van Weeren, P.R.; Hernlund, E. Improving gait classification in horses by using inertial measurement unit (IMU) generated data and machine learning. *Sci. Rep.* **2020**, *10*, e17785, doi:10.1038/s41598-020-73215-9.

Slade, L.M. Conformation and gait characteristics of Icelandic, Tennessee Walker and Walkony horses. In *Proceedings of the Second International Workshop on Animal Locomotion: Fallbrook, California, USA, 12th to 14th March 1993*; Schamhardt, H.C.; Clayton, H.M.; Wade, J.F, Eds.; Equine Veterinary Journal: Newmarket, Suffolk, UK, 1993; p. 8.

Solé, M.; Gómez, M.D.; Galisteo, A.M.; Santos, R.; Valera, M. Kinematic characterization of the Menorca Horse at the walk and the trot: Influence of hind limb pastern angle. *J. Equine Vet. Sci.* **2023**, *33*, 726–732, doi10.1016/j.jevs.2012.12.002.

Solé, M.; Santos, R.; Molina, A.; Galisteo, A.; Valera, M. Genetic analysis of kinematic traits at the trot in Lusitano horse subpopulations with different types of training. *Animal* **2014**, *8*, 192–199, doi:10.1017/S1751731113002036.

Splan, R.K.; Hunter, H.B. Temporal variables of the canter of the Tennessee Walking Horse. *Equine Comp. Exerc. Physiol.* **2004**, *1*, 41–44, doi:10.1079/ECP20033.

Staiger, E.A.; Abri, M.A.; Silva, C.A.S.; Brooks, S.A. Loci Impacting Polymorphic Gait in the Tennessee Walking Horse. *J. Anim. Sci.* **2016**, *94*, 1377–1386; doi:10.2527/jas.2015-9936.

Staiger, E.A.; Bellone, R.R.; Sutter, N.B.; Brooks, S.A. Morphological variation in gaited horse breeds. *J. Equine Vet. Sci.* **2016**, *43*, 55-65, doi:10.1016/j.jevs.2016.04.096.

Starke, S.D.; Clayton. H.M. A universal approach to determine footfall timings from kinematics of a single foot marker in hoofed animals. *PeerJ* **2015**, *3*, e783, doi10.7717/peerj.783.

Starke, S.D.; Robilliard, J.J.; Weller, R.; Wilson, A.M.; Pfau, T. Walk-run classification of symmetrical gaits in the horse: A multidimensional approach. *J. R. Soc. Interface* **2008**, *6*, 335–342, doi:10.1098/rsif.2008.0238.

Stefánsdóttir, G.J.; Jansson, A.; Ragnarsson, S.; and Gunnarsson, V. Speed of gaits in Icelandic horses and relationships to sex, age, conformation measurements and subjective judges' scores, *Comp. Exerc. Physiol.* **2021**, *17*, 151–160, doi:10.3920/CEP200039.

Streitlein, I.; Preuschoft, H. Die Kinematik der Trabtempi von Reitpferden. In *Studien zu den Bewegungen von Sportpferden*; Preuschoft, H., Fritz, M., Huellen-Kluge, K., Knisel, G.,

Streitlein, I., Eds.; Deutsche Reiterliche Vereinigung: Warendorf, Germany, 1987; pp. 20–65; ISBN 978-38-8542-194-8.

Torres-Pérez, Y.; Gómez-Pachón, E.Y.; Miró-Rodríguez, F. Cinemática 2D de caballos al trote mediante videometría y modelamiento matemático. *Rev. Fac. Ing.* **2017**, *26*, 83–96, doi:10.19053/01211129.v26.n45.2017.6057.

Usherwood, J.R. An extension to the collisional model of the energetic cost of support qualitatively explains trotting and the trot–canter transition. *J. Exp. Zool. Part A* **2020**, *333*, 9–19, doi:10.1002/jez.2268.

Van Weeren, P.R.; Van den Bogert, A.J.; Back, W.; Bruin, G.; Barneveld, A. Kinematics of the standardbred trotter measured at 6, 7, 8 and 9 m/s on a treadmill before and after 5 months of pre-race training. *Acta Anat.* **1993**, *146*, 154–161, doi:10.1159/000147438.

Waldern, N.M; Wiestner, T.; Ramseier, L.C.; Weishaupt, M.A. Comparison of limb loading and movement of Icelandic horses while tölting and trotting at equal speeds. *Am. J. Vet. Res.* **2015**, *76*, 1031–1040, doi:10.2460/ajvr.76.12.1031.

Walker, V.A.; Tranquille, C.A.; Newton, J.R.; Dyson, S.J.; Brandham, J.; Northrop, A.J.; Murray, R.C. Comparison of limb kinematics between collected and lengthened (medium/extended) trot in two groups of dressage horses on two different surfaces. *Equine Vet. J.* **2017**, *49*, 673–680, doi:10.1111/evj.12661.

Weishaupt, M.A.; Hogg, H.P.; Auer, J.A.; Wiestner, T. Velocity-dependent changes of time, force and spatial parameters in Warmblood horses walking and trotting on a treadmill. *Equine Vet. J.* **2010**, *42*, 530–537, doi:10.1111/j.2042-3306.2010.00190.x.

Weishaupt, M.A.; Waldern, N.M.; Amport, C.; Ramseier, L.A.; Wiestner, T. Effects of shoeing on intra- and inter-limb coordination and movement consistency in Icelandic horses at walk, tölt, and trot. *Vet. J.* **2013**, *198*, e109–113, doi:10.1016/j.tvjl.2013.09.043.

Willemen, M.A.; Savelberg, H.H.C.M.; Bruin, G.; Barneveld, A. The effect of toe weights on linear and temporal stride characteristics of Standardbred trotters. *Vet. Q.* **1994**, *16*, 97–100, doi:10.1080/01652176.1994.9694511.

Wilson, B.D.; Neal, R.J.; Howard, A.; Groenendyk, S. The gait of pacers 1: Kinematics of the racing stride. *Equine Vet. J.* **1988a**, *20*, 341–346, doi:10.1111/j.2042-3306.1988.tb01542.x.

Wilson, B.D.; Neal, R.J.; Howard, A.; Groenendyk, S. The gait of pacers 2: Factors influencing pacing speed. *Equine Vet. J.* **1988b**, *20*, 347–351, doi:10.1111/j.2042-3306.1988.tb01543.x.

Ziegler, L. *Easy-Gaited Horses*; Storey Publishing: North Adams, Massachusetts, USA, 2005; ISBN 978-15-8017-562-3.

Zyderveld, MC. *De laterale symmetrische gangen, in het bijzonder die van het paard*. Thesis. Rijksuniversiteit Utrecht: Utrecht, The Netherlands, 1991.
